# New Medical Journal in New Orleans

**Published:** 1845-08

**Authors:** 


We have received a circular from New Orleans, by which we are apprised that a new medical Journal is about to be started in that city, under the auspices, as we infer, of the Louisiana Medical College. It is to be edited by Professors *Harrison and Carpenter*, names which bespeak for it the most favorable anticipations.

